# Detection of additional primary malignancies: the role of CT and PET/CT combined with multiple percutaneous biopsy

**DOI:** 10.1590/0100-3984.2018.0024

**Published:** 2019

**Authors:** Tiago Kojun Tibana, Rômulo Florêncio Tristão Santos, Adalberto Arão Filho, Bernardo Bacelar, Leticia de Assis Martins, Rafael Oliveira de Souza, Edson Marchiori, Thiago Franchi Nunes

**Affiliations:** 1 Hospital Universitário Maria Aparecida Pedrossian da Universidade Federal de Mato Grosso do Sul (HUMAP-UFMS), Campo Grande, MS, Brazil.; 2 MS Diagnósticos Médicos, Campo Grande, MS, Brazil.; 3 Universidade Federal de Minas Gerais (UFMG), Belo Horizonte, MG, Brazil.; 4 Instituto de Tratamento do Câncer (ITC), Campo Grande, MS, Brazil.; 5 Universidade Federal do Rio de Janeiro (UFRJ), Rio de Janeiro, RJ, Brazil.

**Keywords:** Neoplasms, second primary/etiology, Biopsy, needle/methods, Positron-emission tomography/methods, Tomography, X-ray computed/methods, Fluorodeoxyglucose F18, Segunda neoplasia primária/etiologia, Biópsia por agulha/métodos, Tomografia por emissão de pósitrons/métodos, Tomografia computadorizada/métodos, Fluordesoxiglicose F18

## Abstract

**Objective:**

To evaluate the imaging findings of ^18^F-fluorodeoxyglucose positron emission tomography/computed tomography (^18^F-FDG PET/CT) and computed tomography (CT) in patients with additional primary tumors, correlating the results with those of the method used in order to elucidate the diagnosis and of the pathology reports.

**Materials and Methods:**

We retrospectively analyzed the medical records, pathology reports and images of 11 patients who underwent CT, ^18^F-FDG PET/CT, or both. We included patients with at least two tumors, with confirmed distinct histopathological profiles, at different sites. Patients in whom there was no diagnostic confirmation were excluded, as were those in whom the additional lesion was suspected of being a metastasis of the first.

**Results:**

New primary malignancies were identified in 11 patients, one new tumor being found in 10 and two new tumors being found in 1. The confirmed sites of the additional malignancies were the lung, kidney, prostate, jejunum, and breast. Single or multiple percutaneous biopsies were performed in 10 patients, and 1 patient underwent a surgical procedure for diagnostic and therapeutic purposes. The tumors were metachronous in 6 cases and synchronous in 5.

**Conclusion:**

CT and ^18^F-FDG PET-CT combined with multiple percutaneous biopsy could facilitate the diagnosis of additional lesions, thus optimizing the treatment and follow-up of the affected patients.

## Abstract (pt)

**Objetivo:**

Avaliar os achados de imagem da tomografia computadorizada por emissão de pósitrons com ^18^F-fluordesoxiglicose (^18^F-FDG PET/TC) e tomografia computadorizada (TC) em pacientes portadores de tumores primários adicionais, correlacionando com o método realizado para elucidação do diagnóstico e relatórios anatomopatológicos.

**Materiais e Métodos:**

Avaliamos, retrospectivamente, prontuários, relatórios anatomopatológicos e exames de 11 pacientes que realizaram ^18^F-FDG PET/TC e/ou TC. Foram incluídos pacientes que apresentaram pelo menos duas neoplasias, com histopatologia distinta confirmada nos diferentes locais. Foram excluídos pacientes sem confirmação diagnóstica e pacientes com suspeita de que a lesão adicional fosse uma metástase da primeira.

**Resultados:**

Lesões sugestivas de novas malignidades primárias foram encontradas em 11 pacientes, apresentando em 10 deles uma única nova lesão e em 1 caso dois novos tumores. Locais comprovados de lesão adicional foram pulmão, rim, próstata, jejuno e mama. Biópsia percutânea única ou múltipla foi realizada em 10 pacientes e 1 paciente foi submetido a procedimento cirúrgico para fins diagnósticos e terapêuticos. Os tumores eram metacrônicos em 6 casos e sincrônicos em 5 pacientes.

**Conclusão:**

A TC e a ^18^F-FDG PET/TC associadas a biópsias percutâneas múltiplas podem auxiliar no diagnóstico de lesões adicionais, otimizando o tratamento e acompanhamento desses pacientes.

## INTRODUCTION

Multiple primary tumors can be defined as more than one, histologically different, synchronous or metachronous lesion in the same individual^(^^1^^)^. Although uncommon, their incidence and prevalence have been progressively increasing, due in large part to the increase in life expectancy of the population and to advances in diagnostic techniques^(^^2^^)^. From a diagnostic point of view, their early recognition and confirmation are essential to achieving the ideal treatment. Therefore, radiologists should be familiar with the different patterns of presentation in patients with multiple primary tumors^(^^2^^)^.

Conventional imaging modalities, including ultrasound, computed tomography (CT), and magnetic resonance imaging (MRI), have limitations in the detection of multiple tumors, due to their regional imaging standard. In recent years, ^18^F-fluorodeoxyglucose positron emission tomography/computed tomography (^18^F-FDG PET/CT) has emerged as a promising imaging modality in the evaluation of malignant tumors^(^^3^^)^. In addition, studies indicate that the introduction of ^8^F-FDG PET/CT images to evaluate malignant tumors results in better detection of multiple primary tumors^(^^4^^,^^5^^)^.

The advent of interventional radiology made possible notable advances in the diagnosis and treatment of various situations. Because of ongoing improvements in imaging methods and the need to seek more effective and less aggressive treatments, image-guided procedures performed by interventional radiologists have taken on added importance in the field of oncology^(^^6^^)^. The objective of this study was to evaluate the imaging findings of ^18^F-FDG PET/CT and CT in patients with additional primary tumors, correlating the results with those of the method used in order to elucidate the diagnosis and of the pathology reports.

## MATERIALS AND METHODS

We retrospectively analyzed the images acquired at two imaging and interventional radiology clinics, one at a public hospital and another at a private clinic, between January 2016 and January 2018. Clinical data were obtained from medical records and through telephone contact with physicians, patients, and family members. We selected patients who had presented with histologically proven synchronous or metachronous primary tumors and had undergone CT and/or PET/CT for either diagnostic or follow-up purposes. The inclusion criterion was the presence of at least two neoplasms, confirmed by histopathological examination, with distinctive histopathology at the different sites. We used an interval of six months to differentiate between synchronous and metachronous neoplasms, a criterion previously used by several other authors^(^^7^^-^^9^^)^. Patients in whom there was no diagnostic confirmation were excluded, as were those in whom the additional lesion was suspected of being a metastasis of the first. The final sample comprised 11 patients (8 males and 3 females). The previously recognized primary tumors and the suspicion of the new primary site were recorded, as well as their histological classification. Percutaneous procedures guided by CT are safe, well-established techniques. Biopsies guided by functional studies, such as PET/CT, have been widely studied in the literature. For each case, we analyzed the procedure performed by interventional radiology in order to elucidate the diagnosis, as well as other treatments and the outcome.

All examinations were performed in a 128-slice multidetector PET/CT scanner (Discovery 610; General Electric, Milwaukee, WI, USA), after a ≥ 6-h fast. The patients received an intravenous solution of ^18^F-FDG, with an activity count of 3.7-5.2 MBq/kg (0.10-0.14 mCi/kg). The PET/CT images were acquired after 60 to 120 minutes. Post-processing was performed with multiplanar reconstructions and maximum intensity projection techniques.

A radiologist with seven years of experience in abdominal diagnostic imaging and a nuclear physician with ten years of experience in PET/CT examinations, together with two radiology residents, analyzed the imaging studies, determining the quantity and location of the lesions. In addition, clinical records, pathology reports, and outcomes were analyzed. Details such as patient age at the time of diagnosis of each tumor, patient gender, tumor type (metachronous or synchronous), tumor site of origin, diagnostic method, histological classification, and treatment regimen were all recorded. All data were tabulated and analyzed in a Microsoft Excel spreadsheet.

## RESULTS

**Figure 1:**
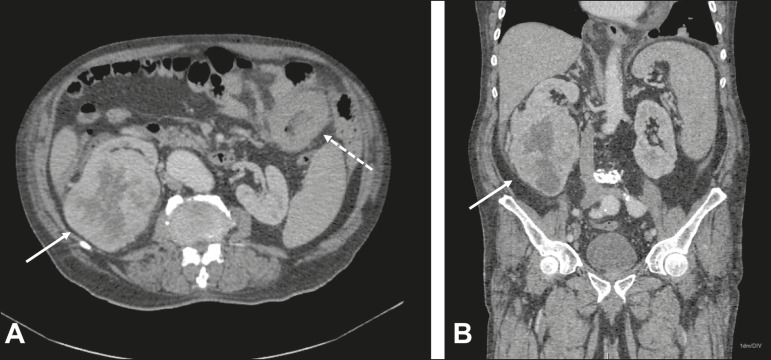
60-year-old male undergoing restaging of a previously resected squamous cell carcinoma of the penis. Axial and coronal CT (**A** and **B**, respectively) showing multiple pulmonary nodules, a mass in the right kidney (arrows), a lesion in the jejunum (dashed arrow), and inguinal lymph node enlargement on the right. A percutaneous biopsy confirmed primary adenocarcinoma of the jejunum with pulmonary and inguinal metastases and clear cell carcinoma in the right kidney.

**Figure 2:**
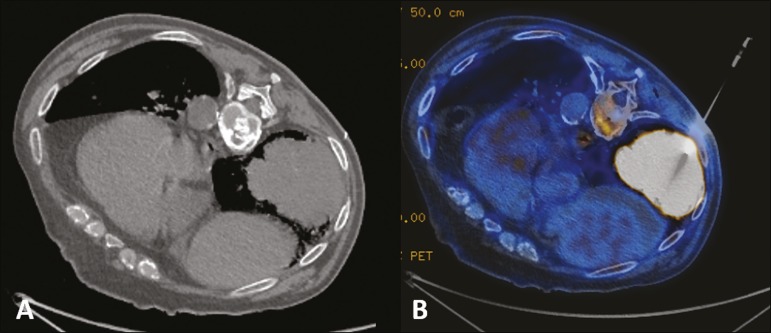
56-year-old male patient. Axial CT scan showing a voluminous lesion in the lower lobe of the left lung (**A**). Biopsy guided by ^18^F-FDG PET/CT showed homogeneous uptake, with no areas of necrosis, increasing the diagnostic accuracy of the procedure and revealing adenocarcinoma (**B**).

**Figure 3:**
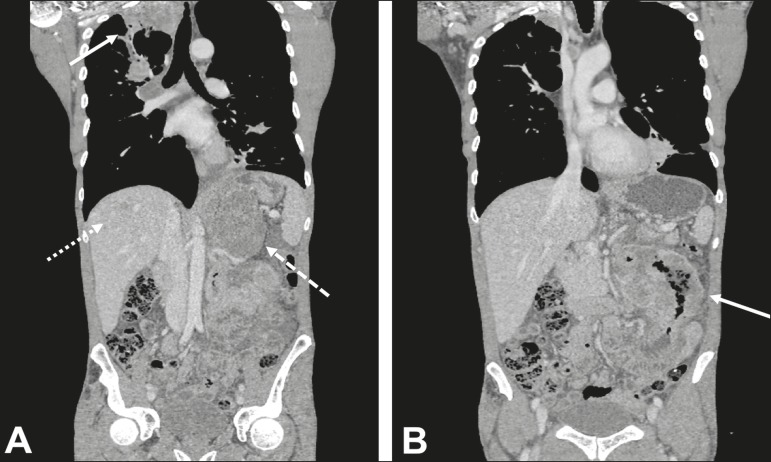
62-year-old male with recurrent hemoptysis, weight loss, and abdominal pain. **A:** CT showing a cavitary pulmonary lesion (arrow), a right adrenal mass (dashed arrow), and a hepatic nodule (dotted arrow). **B:** Asymmetrical circumferential thickening of the jejunum (arrow). Pathology report after percutaneous biopsy confirmed adenocarcinoma of the jejunum and squamous cell carcinoma of the lung with metastases to the adrenal glands and liver.

**Figure 4:**
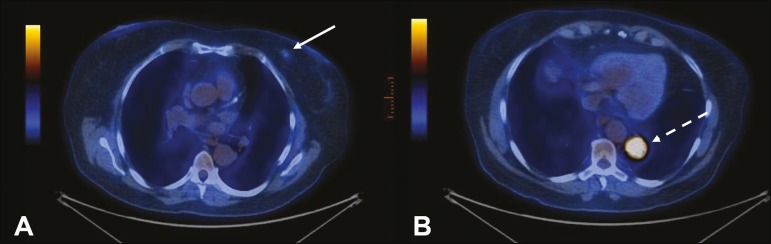
71-year-old female under investigation for a chronic cough. ^18^F-FDG PET/CT showed a focal mass in the left breast (arrow in **A**), confirmed as a spiculated nodule in the ultrasound and a lesion with avid uptake in the right lower lobe of the left lung (dashed arrow in **B**). Percutaneous biopsy confirmed invasive ductal carcinoma in the left breast and primary adenocarcinoma of the lung.

The age of the patients ranged from 52 to 80 years. New primary malignancies were identified in 11 patients, one new tumor being found in ten and two new tumors being found in one. The confirmed sites of the additional malignancies were the lung (n = 4), kidney (n = 3), prostate (n = 2), jejunum (n = 2), and breast (n = 1). In all patients, histology and immunohistochemistry showed that the new lesions were clearly different (i.e., primary) malignancies, rather than metastases of the previously recognized primary lesion. In one patient with a previously recognized neoplasm of the penis, a CT scan showed two new lesions, one a clear cell renal carcinoma and another an adenocarcinoma of the jejunum (Figure 1). Ten patients had single or multiple percutaneous biopsies, guided either by ultrasound or CT, and one patient underwent a surgical procedure for diagnostic and therapeutic purposes. Of the 12 new tumors found, six were synchronous and six were metachronous. Among the suspected or previously recognized malignancies, there were two hepatocellular carcinomas; three adenocarcinomas of the lung (Figure 2), two of which had metastasized to the mediastinum, adrenal glands, and liver (Figure 3); one thymoma; one renal clear cell carcinoma with metastasis to the ipsilateral adrenal gland; one squamous cell carcinoma of the penis; one invasive ductal breast carcinoma (Figure 4); one adenocarcinoma of prostate; and one squamous cell carcinoma of the esophagus (Table 1).

**Table 1:** Pathology-confirmed tumors, diagnostic procedures, additional primary malignancies, and outcomes.

	Age					Synchronous/	
Patient	(years)	Gender	Known or suspected tumor	Diagnostic procedure	Additional primary malignancy	Metachronous	Outcome
1	80	Male	Hepatocellular carcinoma	Percutaneous biopsy	Adenocarcinoma of the prostate	Metachronous	Ablation / prostatectomy
2	56	Male	Adenocarcinoma of the lung	Percutaneous biopsy	Renal clear cell carcinoma	Metachronous	Nephrectomy / chemotherapy and radiotherapy of the lung
3	61	Male	Thymoma	Percutaneous biopsy	Adenocarcinoma of the prostate	Metachronous	Thymectomy / chemotherapy
4	68	Male	Renal clear cell carcinoma	Percutaneous biopsy	Renal oncocytoma	Synchronous	Chemotherapy
5	60	Male	Squamous cell carcinoma of the penis	Surgery	Renal clear cell carcinoma/ adenocarcinoma of the jejunum	Synchronous	Death
6	62	Male	Adenocarcinoma of the lung with adrenal and liver metastases	Percutaneous biopsy	Adenocarcinoma of the jejunum	Synchronous	Chemotherapy
7	71	Female	Adenocarcinoma of the lung	Percutaneous biopsy	Invasive ductal cell carcinoma	Synchronous	Hormone therapy / resection of the pulmonary lesion
8	52	Female	Invasive ductal breast carcinoma	Percutaneous biopsy	Squamous cell carcinoma of the lung	Metachronous	Chemotherapy
9	69	Female	Hepatocellular carcinoma	Percutaneous biopsy	Adenocarcinoma of the lung	Metachronous	Transplant / chemotherapy and radiotherapy
10	66	Male	Adenocarcinoma of the prostate	Percutaneous biopsy	Adenocarcinoma of the lung	Metachronous	Prostatectomy / transferred
11	58	Male	Squamous cell carcinoma of the esophagus	Percutaneous biopsy	Adenocarcinoma of the lung	Synchronous	Transferred

Treatments ranged from chemotherapy alone (in three cases); chemotherapy and surgical resection (in one case); chemotherapy and radiotherapy with surgical resection (in one case); hormone therapy with surgical resection (in one case); ablation of hepatocellular carcinoma and surgical resection (in one case); and transplantation combined with chemotherapy and radiotherapy (in one case). Only one patient progressed to death, three months after the diagnosis of new lesions. Two patients were transferred to other centers for monitoring and treatment, one of them undergoing surgical resection prior to the transfer (Table 1).

## DISCUSSION

Multiple primary tumors can be defined as more than one synchronous or metachronous lesion in the same individual. By definition, multiple primary tumors are histologically different, involving different organs, and metastatic lesions are excluded. Two tumors are categorized as synchronous when they correspond to another tumor site in the same patient and are diagnosed within six months of each other, whereas the second tumor would be categorized as metachronous if diagnosed more than six months after the diagnosis of the index tumor^(^^1^^)^.

The risk of developing a new primary tumor is 20% higher in patients with an existing neoplasm than in the general population^(^^10^^)^. Approximately one third of cancer patients over the age of 60 are diagnosed with an additional primary lesion. The most common risk factors are genetic predisposition, lifestyle, hormonal imbalance, environmental exposures, and previous treatment of a primary tumor^(^^11^^)^.

Some types of neoplasms tend to be grouped by the risk factors they share: smoking in cancers of the lung, head, and neck; dietary or endocrine factors in gynecological cancers; ultraviolet light in melanoma and skin cancer; and viral agents in cervical and anogenital cancers. Subsequent additional primary malignancies may also be associated with a potentially carcinogenic treatment of the initial lesion, such as chemotherapy, radiotherapy, or both. In addition, genetic risk factors such as BRCA1 and BRCA2 mutations have been linked to a predisposition to multiple malignancies, such as breast cancer and ovarian cancer^(^^12^^)^.

Genetic conditions can trigger the hereditary cancer syndromes that are characterized by a higher prevalence of neoplasia in individuals of the same family^(^^13^^)^ and a high risk of developing tumors at an early age, as well as multiple primary tumors, either metachronous or synchronous^(^^14^^)^. Knowledge of these syndromes is important, because the initial diagnosis of an "index" tumor can prompt the investigation of a possible syndromic context and the discovery of other lesions. One example is a diagnosis of hemangioblastoma of the central nervous system, leading to early screening for von Hippel-Lindau syndrome^(^^15^^)^; a diagnosis of pulmonary hamartoma, in the context of the Carney triad^(^^16^^)^; or a diagnosis of medullary thyroid carcinoma in young patients, in the context of multiple endocrine neoplasia^(^^17^^)^.

In other, more common, tumors, such as colorectal carcinoma, endometrial carcinoma, and sebaceous neoplasms of the skin, a study of the immunohistochemistry of the lesions can be requested to evaluate any microsatellite instability (DNA mismatch repair) or possible association with Lynch syndrome or Muir-Torre syndrome^(^^18^^)^.

The detection of unexpected malignant lesions has a significant clinical impact, not only on healthy individuals but also on patients with previously recognized malignant disease. Studies involving patients with previously diagnosed tumors typically focus on the primary disease, and the incidental coexistence of another primary malignant tumor can therefore be missed^(^^19^^)^. From a diagnostic point of view, the early recognition and confirmation of such tumors are essential to determining the ideal treatment. Therefore, radiologists should be familiar with the different patterns of presentation in a patient with multiple primary tumors^(^^2^^)^.

PET with ^18^F-FDG is being used with increasing frequency in the evaluation and clinical management of an ever greater number of neoplasms^(^^20^^-^^23^^)^. Some reports also indicate that PET with ^18^F-FDG has potential as a screening method for cancer and can detect new malignant tumors that other imaging methods fail to detect in asymptomatic individuals^(^^24^^,^^25^^)^. The PET/CT combination is a promising hybrid imaging modality that is being used routinely in different clinical situations^(^^26^^-^^29^^)^, because it allows precise integration of metabolic PET images with high-quality CT images in an acquisition that goes from the top of the head to the upper thigh^(^^19^^)^. FDG is a glucose analog used as a marker with fluorine-18, a positron-emitting radioisotope, which allows the study of the glucose metabolism because of its higher uptake by tissues with increased glycolysis. A broad spectrum of biochemical changes is present in tumor cells, including higher rates of aerobic and anaerobic glycolysis when compared with those found in normal tissues. The location of ^18^F-FDG absorption can be precisely determined based on these images, which highlight the metabolic differences between benign and malignant cells^(^^19^^,^^21^^,^^30^^)^.

CT-guided biopsy has been widely used as an effective, safe procedure for diagnostic confirmation in various clinical contexts. A biopsy guided by PET/CT, which combines anatomical information obtained from CT and metabolic information from PET with ^18^F-FDG, is a procedure that can optimize the diagnostic yield of image-guided interventions, given that lesions presenting uptake of ^18^F-FDG, without a corresponding anatomical anomaly, may be accessible to percutaneous interventions^(^^31^^)^. Although there have been no studies demonstrating any significant differences between PET/CT and CT in terms of their ability to obtain a viable biopsy sample or their complication rates, we believe that PET/CT is a particularly important method, given that CT has been shown to fail to detect lesions that later appear as foci of avid ^18^F-FDG uptake^(^^31^^)^.

Image-guided percutaneous biopsy is a well-established and safe technique and plays a crucial role in the management of oncology patients. Improvements in the design of needles, the development of new biopsy techniques, and ongoing technological advancements in image orientation have improved the safety and effectiveness of the procedures. Lesions that were previously considered relatively inaccessible can now be accessed safely^(^^32^^)^. Some of the advantages of interventional radiology include the possibility of performing complex procedures with small incisions, decreasing the likelihood of infections, promoting the rapid recuperation of the patient, and reducing hospitalization time, given that the techniques involved are minimally invasive, safe, and highly effective^(^^6^^)^.

## CONCLUSION

^18^F-FDG PET/CT can be used as a tool to complement imaging methods such as CT^(^^30^^)^ and, when combined with minimally invasive procedures in interventional radiology, can be useful in the identification of additional primary malignancies the early recognition and diagnosis of which are essential, because the management of cases is often changed if this information is available. Studies show that, although false-positives can occur, the prevalence of true-positives is substantial^(^^19^^)^. Additional primary malignancies are often identified in the initial phase, and the chance of a cure is therefore excellent if such malignancies are treated promptly and aggressively^(^^19^^)^. Biopsies guided by ^18^F-FDG PET/CT can help in difficult situations, especially when it is important to know which part of the tumor is active or which lesion is active in patients with multiple, disseminated lesions^(^^33^^)^.
